# The Stoppa combined with iliac fossa approach for the treatment of both-column acetabular fractures

**DOI:** 10.1186/s13018-020-02133-3

**Published:** 2020-12-07

**Authors:** Yun Yang, Chang Zou, Yue Fang

**Affiliations:** Department of Orthopaedics, West China Hospital, Sichuan University, Chengdu, Sichuan People’s Republic of China

**Keywords:** Acetabular fracture, Both columns, Stoppa approach, Iliac fossa approach, Ilioinguinal approach, Internal fixation

## Abstract

**Background:**

At present, the choice of surgical approach for both-column fractures is still controversial. The purpose of this study was to explore the efficacy of the Stoppa combined with iliac fossa (S+IF) approach in the treatment of both-column fractures.

**Methods:**

In this retrospective case series, 76 patients were included in the study from 2014 to 2018. They were divided into two groups according to the surgical approaches. The differences of intraoperative blood loss, operative time, quality of reduction, clinical outcome, and perioperative complications were compared between the two groups.

**Results:**

All patients had undergone the IL approach or the S+IF approach. The average operative time was 156.2 min (110~210 min) in group I and 126.5 min (80~180 min) in group II (*P* < 0.001). The average blood loss in group I was 784.1 ml, while the average blood loss in group II was 625.3 ml (*P* = 0.007). According to Matta’s criteria, 28 cases obtained anatomic reduction and 12 cases got imperfect reduction in group I; 21 cases obtained anatomic reduction and 7 cases got imperfect reduction in group II (*P* > 0.05). The clinical outcome (excellent to good) was 66% in group I versus 69% in group II (*P* > 0.05). The complication rates were 18.2% in group I and 12.5% in group II (*P* > 0.05)*.*

**Conclusions:**

As a minimally invasive surgical approach, the S+IF approach is a valuable alternative to the IL approach for the treatment of both-column acetabular fractures if these two anterior approaches can achieve fracture exposure, reduction, and fixation.

## Background

Both-column fractures account for about 20% of the total number of acetabular fractures, which are characterized by no articular fragment in connection with the axial skeleton and fracture lines involving multiple planes [1–3]. In the treatment of acetabular fractures, anatomical reduction and rigid internal fixation are very critical to obtain a good outcome [3–6]. In order to achieve accurate reduction and minimize complications, it is necessary to choose the appropriate surgical approach.

The choice of surgical approach for both-column fractures is still controversial. The IL approach is the standard anatomic approach for most both-column fractures [7] and enables direct visualization of the anterior column up to the symphysis pubis. The exposure of the quadrilateral plate via the IL approach can only be achieved by palpation of the endopelvic finger. Some scholars actively explore a single approach to treat this complex injury [8–11]. Others are skeptical that neither the IL nor the Kocher-Langenbeck (KL) approach alone can expose all of the fragments [12–14]. However, extensile approaches are associated with higher rates of complications [13–15]. Therefore, limited exposure is necessary to optimize treatment and reduce complications. The Stoppa approach, as a midline approach, can provide direct exposure of the area from the sacroiliac joint to the pubic symphysis (including the quadrilateral plate) [6, 16]. However, it does not expose the iliac wing and is sometimes combined with the lateral window of the IL approach to treat complex acetabular fractures [17, 18].

To overcome the respective limitations of the IL and Stoppa approaches, we have treated complex acetabular fractures through the S+IF approach in recent years. Moreover, there is little literature on the treatment of both-column fractures by S+IF approach. Therefore, the aim of this study was to investigate the clinical efficacy of the S+IF approach in the treatment of both-column fractures by comparing with the IL approach.

## Materials and methods

### Materials

From January 2014 to January 2018, we retrospectively studied 98 patients with acetabular fractures. Inclusion criteria were (1) acute both-column fracture and (2) fractures treated with the S+IF or IL surgical approach. The exclusion criteria were (1) fractures treated via the combined anterior and posterior approaches, (2) preoperative range of motion (ROM) deficiency of the hip, (3) open fractures, (4) less than 1 year of follow-up, (5) conservative treatment, and patients with a history of bladder surgery, hysterectomy, cesarean section, or prostatectomy. Overall, 76 patients met the criteria for inclusion. Data were collected through an anonymous way because the patients’ identifiers such as name and unique identity were erased. This study was approved by the Ethics Committee and Institutional Review Board of West China Hospital.

All patients were studied with X-rays (anteroposterior and Judet oblique views) and CT scan with 3D reconstruction for classification of acetabular fractures. Preoperative skeletal traction was conducted on the affected side to guard against further injury to the femoral head. All patients were treated by the same medical team.

These patients were divided into two groups according to the surgical approaches. The first group included 44 patients treated by the IL approach (group I), while the second group of 32 patients were treated by the S+IF approach (group II).

### Surgical technique

#### IL approach

**Fig. 1:**
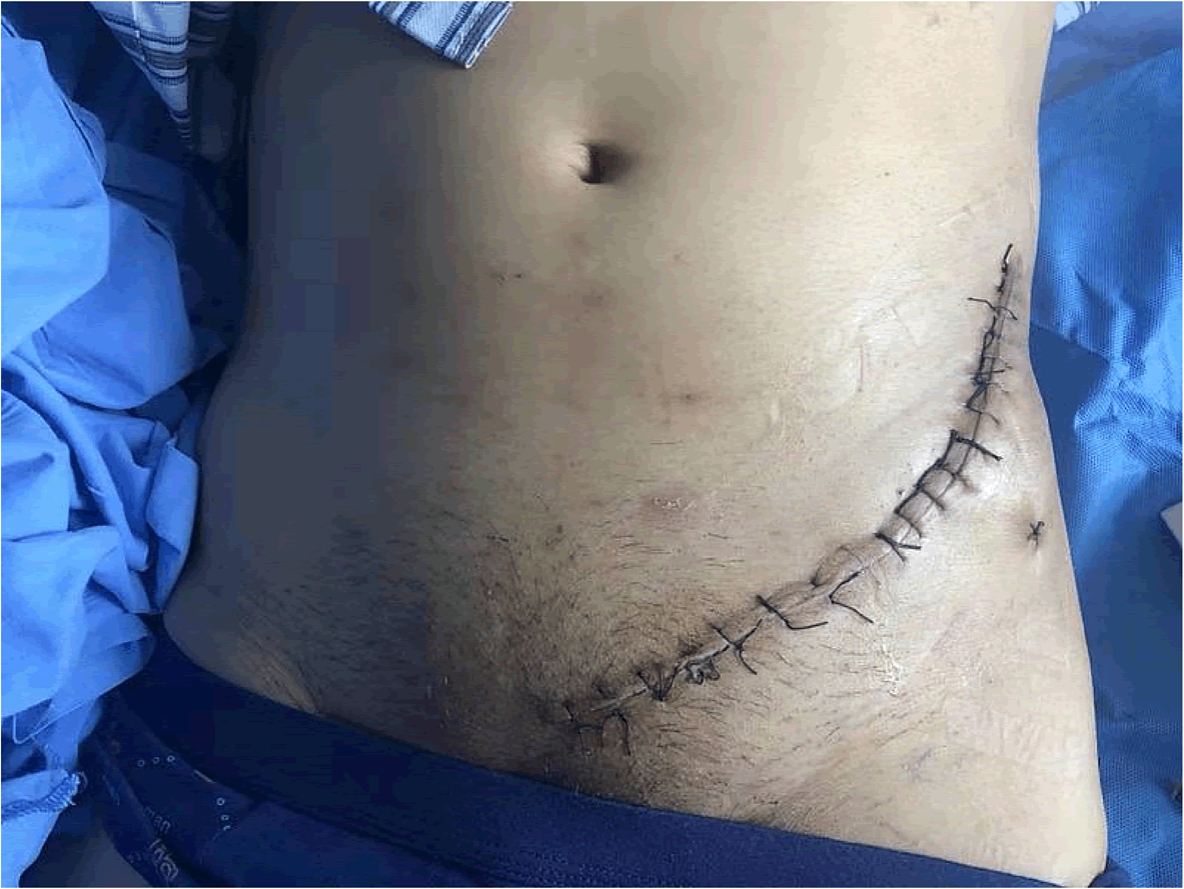
Surgical incision of the IL approach

The incision of the IL approach began at the middle of iliac crest and was anteriorly and distally extending to the anterior superior iliac spine (ASIS) and the pubic tubercle (Fig. 1). The IL approach was divided into three windows by lacuna vasorum and lacuna musculorum. The lateral window could be exposed by subperiosteal dissection of the abdominal muscle and iliac muscle. The aponeurosis of the external oblique was carved to identify and dissociate the external ring of inguinal canal and expose the medial window. The aponeurosis of the internal oblique was incised, followed by a wide drainage film to free and protect the femoral vessels, femoral nerve, lymphatic vessels, and conjunctive tendon. Finally, the iliopubic fascia was separated and cut to expose the middle window. After satisfactory reduction was accomplished, a prebent plate was placed for fixation.

#### S+IF approach

**Fig. 2:**
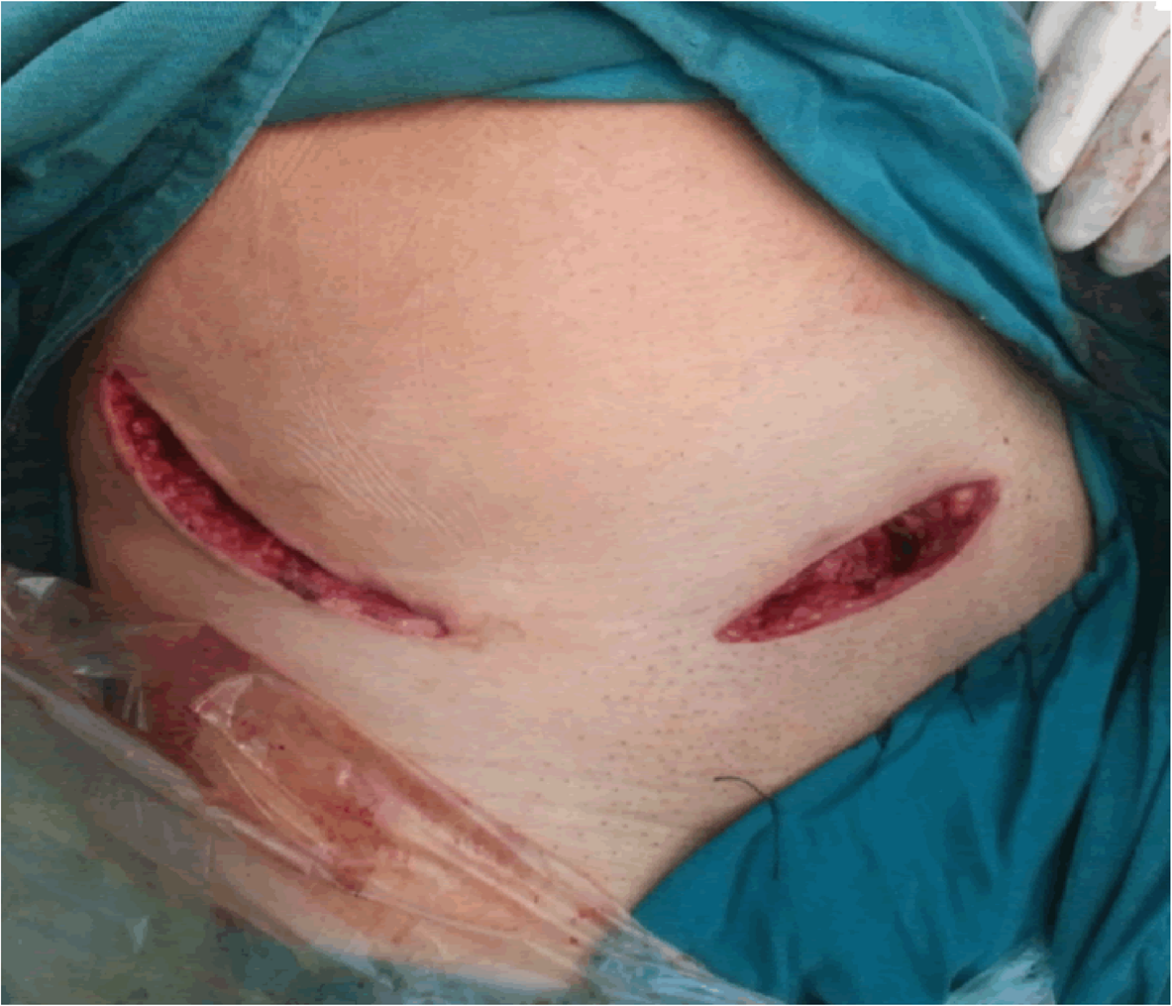
Surgical incision of the S+IF approach

The patient who received the S+IF approach (Fig. 2) was placed in the supine position. The incision of the Stoppa approach was performed in the region 1~2 cm above the pubic symphysis. The bladder was protected, and the rectus abdominis was retracted to expose the pubic symphysis and the superior ramus of the pubis. The rectus abdominis and neurovascular bundle were then retracted laterally to protect them. If corona mortis was encountered, ligation should be performed. The iliopubic fascia and obturator fascia were incisively dissected from the front to the back to expose the true pelvic rim, the quadrilateral plate, and the posterior column of the acetabulum. The ischial support band and the back of the pelvic rim could be better exposed by stripping the psoas muscle. The IF approach (the lateral window of the IL approach) began at the middle of the iliac crest to the ASIS. The exposure of the iliac wing could be achieved by retracting the iliac muscle and iliac vessels, and the exposure could be improved by flexion, rotation, adduction, and abduction of the affected hip. Reduction techniques included the use of a ball-spiked pusher to provide an outward force, the insertion of a Hohmann lever at the greater sciatic notch to pry out the posterior column and femoral traction, or the use of traction bed for longitudinal traction.

### Follow-up and evaluation criteria

The patients’ charts were surveyed for intraoperative blood loss, operative time, quality of reduction, clinical outcome, and perioperative complications. The quality of the reduction was evaluated based on the immediately postoperative radiographs. It was graded as anatomic, imperfect, and poor according to the scoring system published by Matta [4].

Follow-up was performed at 1, 2, and 3 months and 1 year after surgery and yearly thereafter. Clinical outcomes were measured using the Matta modification of the Merle D’Aubigne score [7, 19]. Complications such as infection, neurovascular injuries, and hematoma were also recorded.

### Statistical analysis

Statistical analysis was done using the SPSS 20.0 software (SPSS Chicago, IL, USA). The results were presented as the mean ± standard deviation. The complication rates of two groups were determined by chi-square test. Other differences between the two groups were determined by *t* tests. A value of *P* ≤ 0.05 was considered to be statistically significant.

## Results

### The demographics of subjects

All fractures were fixed via the IL approach (44 cases) or the S+IF approach (32 cases). There were 47 (62%) males and 29 (38%) females included in this study with an average age of 41 years (range 18~75 years). The most common mechanism of injury was a high-energy mechanism—fall from height. The mean follow-up was 26 months (range 12~36 months). The difference of general characteristics between the two groups was not statistically significant (Table 1).

**Table 1:** The demographics of subjects

Variable	Group		*P* value
	I	II	
Age (M ± SD), year	41.89 ± 14.19	39.94 ± 15.21	0.405
Gender, *n* (%)			
Men	27 (61)	20 (62)	0.920
Women	17 (39)	12 (38)	
Side of injury, *n* (%)			
Right	26 (59)	15 (47)	0.291
Left	18 (41)	17 (53)	
Bilateral	0 (0)	0 (0)	
Mechanism of injury, *n* (%)			
Motor vehicle collision	15(34)	12 (38)	0.763
Fall from height	26 (59)	19 (59)	
Others	3 (7)	1 (3)	
Total	44	32	–

### Comparison of surgical-related parameters

The average blood loss in group I was 784.1 ml, while the average blood loss in group II was 625.3 ml (*P* = 0.007). The mean operative time was longer in group I as compared to group II (*P* < 0.001). There was no secondary congruence for the reduction in this study. The quality of reduction was similar between the two groups (*P* = 0.806). The clinical outcome (excellent to good) was 66% in group I versus 69% in group II, and the difference was not statistically significant (Table 2).

**Table 2:** Analysis of the results between the two groups

Variable	Group		*P* value
	I	II	
Blood loss (ml)	784.09 ± 277.70	625.31 ± 193.39	0.007
Operative time (min)	156.18 ± 27.54	126.53 ± 29.56	< 0.001
Reduction (mm)			0.806
Anatomic (0–1)	28 (64%)	21 (66%)	
Imperfect (2–3)	12 (27%)	7 (22%)	
Poor (> 3)	4 (9%)	4 (12%)	
Clinical outcome, score			0.981
Excellent (15–18)	21 (48%)	15 (47%)	
Good (11–14)	8 (18%)	7 (22%)	
Fair (7–10)	9 (20%)	6 (19%)	
Poor (< 7)	6 (14%)	4 (12%)	

### Comparison of complications

The complication rates were 18.2% in group I (8 cases) and 12.5% in group II (4 cases) (*P* = 0.502) (Table 3). There were three cases of lateral femoral cutaneous nerve (LFCN) palsy with 3~6 months resolution in group I. Femoral nerve palsy developed in one patient, and this case showed part recovery after 1 year. Femoral vascular injury occurred in one patient. Hematoma and surgical wound infection appeared in one and two patients, respectively, with the symptoms disappearing after conservative treatment (puncture and antibiotics, respectively). In group II, LFCN palsy existed in two cases postoperatively, and the symptoms disappeared after 4 months of nutritional neurotherapy. The obturator nerve was damaged in one case intraoperatively but had recovered 6 months after surgery. One patient had an iatrogenic laceration of the corona mortis during surgery. Nonunion heterotopic ossification was not observed in this study.

**Table 3:** Comparison of perioperative complications between the two groups

Complications	Group		*P* value
	I	II	
LFCN palsy	3	2	0.502
ON palsy	0	1	
FN palsy	1	0	
Vascular injury	1	1	
Hematoma	1	0	
Infection	2	0	
DVT	0	0	
Total	8	4	

*LFCN* lateral femoral cutaneous nerve, *ON* obturator nerve, *FN* femoral nerve, *DVT* deep vein thrombosis

## Discussion

Surgical approach is the key factor affecting the treatment effect of acetabular fractures [20]. The IL approach enables a wide view of the entire iliac surface, the iliac crest, and the sacroiliac joint anteriorly [21]. Its indications include fractures of the anterior wall, the anterior column, anterior column plus posterior hemitransverse, some T types, and most of the associated both-column fractures [7]. However, it just gives palpatory exposure of the quadrilateral plate and enables indirect exposure of the posterior column within the middle window. In addition, the learning curve is quite steep due to critical anatomical structures. The Stoppa approach has been modified for the treatment of acetabular fractures. It can well expose the pelvic ring and the quadrilateral plate, which facilitates the fracture reduction at the quadrilateral plate. But the single Stoppa approach does not adequately expose and fix the iliac wing fracture, which could be exposed through the IF approach.

Considering the defects of the above surgical approaches, we combined the S+IF approach to treat acetabular fractures. There were few reports on the treatment of both-column fractures by the S+IF approach. Therefore, we compared the efficacy of the IL approach and the S+IF approach in the treatment of both-column fractures.

Compared with the IL+KL approach, the S+IF approach was less invasive and has less bleeding and less surgical time [22]. Ma et al. [23] found that the Stoppa approach reduced intraoperative blood loss and there were no significant differences in other measured variables by comparing the patients’ demographics and the perioperative parameters between the Stoppa approach and the IL approach. Shazar et al. [24] found that the Stoppa approach was superior to the IL approach in terms of reduction accuracy. Rocca et al. [25] found the Anterior Combined Endopelvic (the Stoppa approach with the lateral approach to the iliac crest) approach and the IL approach were similar in reduction quality, while the ACE approach was more effective than the IL approach in clinical outcome and blood loss.

**Fig. 3:**
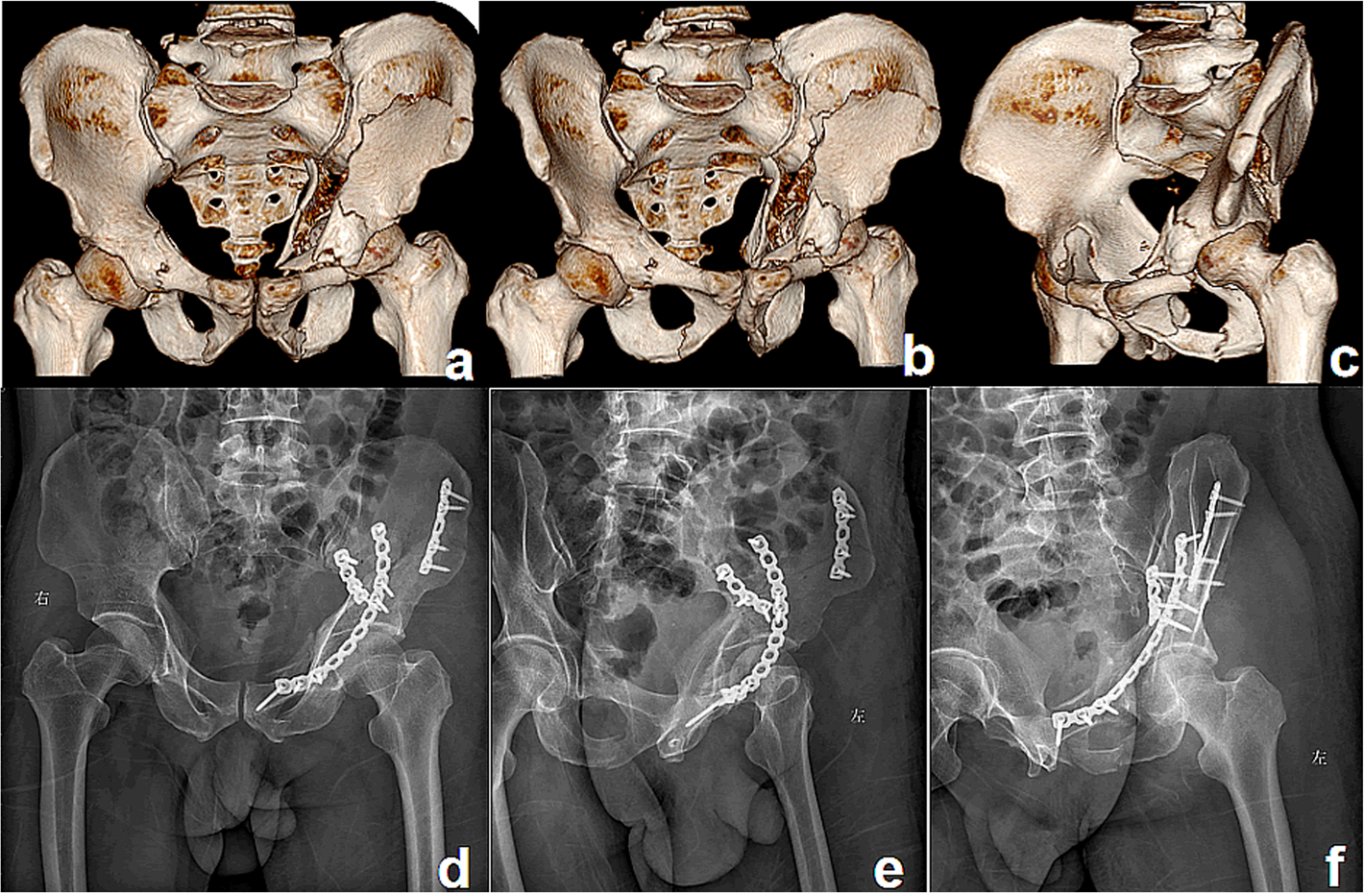
A patient treated with the IL approach. Preoperative anteroposterior (**a**), iliac oblique (**b**), and obturator oblique (**c**) 3D computed tomography reconstructions of a both-column fracture showing the severity of fracture displacement. Postoperative radiographs (**d**–**f**) showing good reduction and fixation

**Fig. 4:**
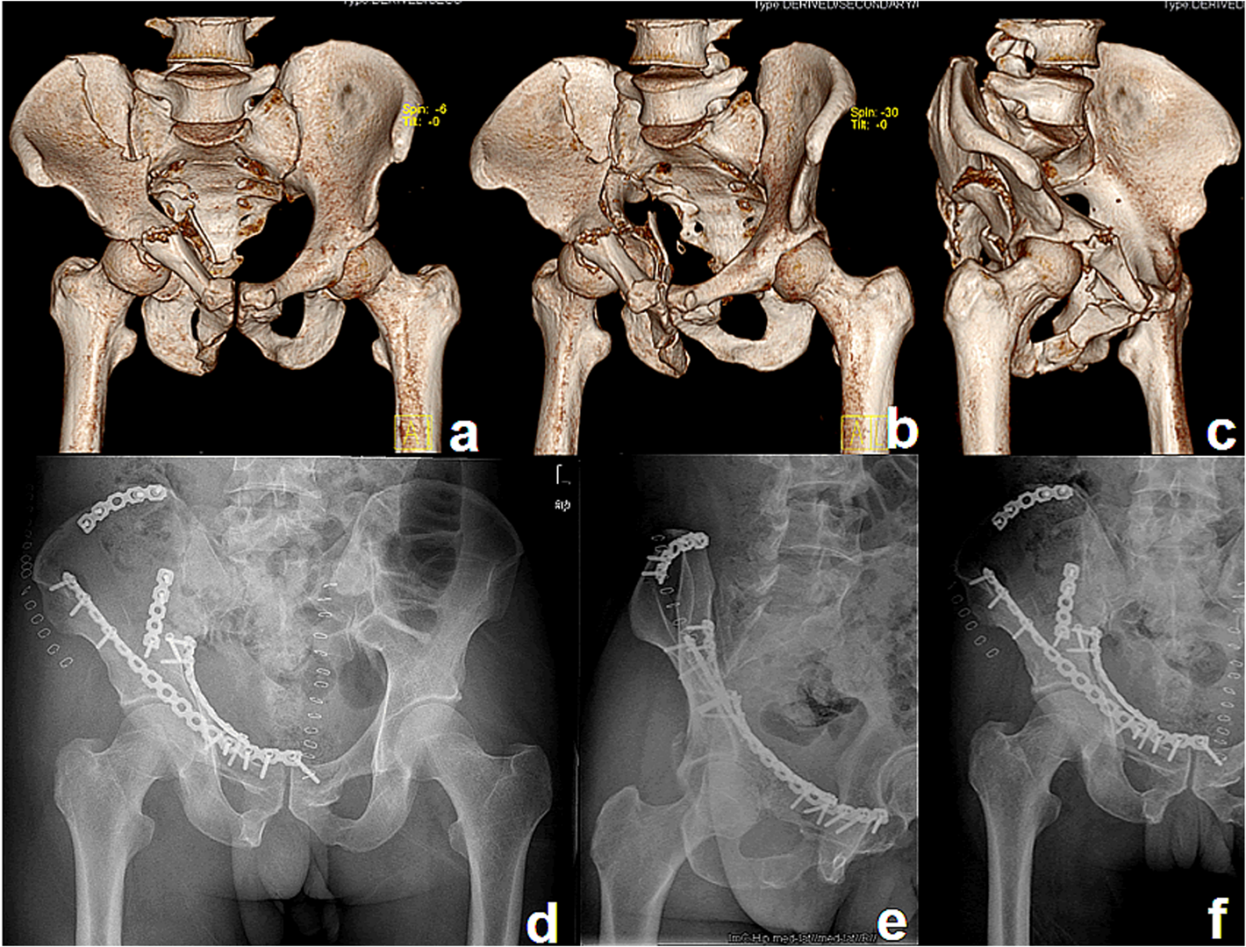
A patient treated with the S+IF approach. Preoperative 3D computed tomography reconstructions (**a**–**c**) of a both-column fracture showing the severity of fracture displacement. Postoperative radiographs (**d**–**f**) showing good reduction and fixation

In the current study, fracture reduction was acceptable in 91% of cases (64% anatomical, 27% imperfect) in group I (Fig. 3) and in 88% of cases (66% anatomical, 22% imperfect) in group II (Fig. 4). The clinical outcome was similar between the two groups. Blood loss and operative time were significantly more in group I than in group II. These results were basically consistent with the above studies [22–25].

The overall rate of complications in our study was comparable to that found in the literature. Most scholars reported similar complications in their early cases [7, 26, 27]. Both the IL and Stoppa approaches were at risk of injury to blood vessels and nerves. LFCN was prone to iatrogenic injury due to its highly variable course and branches [28]. LFCN injury occurred in three patients in group I and two patients in group II. Our experience is that the separation of soft tissue along the iliac periosteum may be conducive to protect LFCN. In addition, vascular injury (femoral vascular versus corona mortis) and other nerve injury (femoral versus obturator) occurred in both groups. When corona mortis is encountered, ligation is recommended to avoid affecting the visibility of the fracture fissure. In this study, wound infection occurred in two patients (2.6%) in group I, with wound healing after intensive dressing change and antibiotic use. But it is not clear whether there is a direct link between wound infection and surgical approach.

This study had also several limitations. As a retrospective study, patients were not randomly assigned. In addition, there were some shortcomings in this study, such as small sample size and short follow-up time, which need to be further improved to confirm the advantages of the S+IF approach in future research.

## Conclusions

In conclusion, the S+IF approach can fully expose the anterior column and the quadrilateral plate, which is conducive to the reduction and fixation of both-column fractures. Compared with the IL approach, the S+IF approach had the advantages of less blood loss and shorter operative time. In terms of reduction accuracy, clinical outcome, and fewer complications, there was no significant difference between the two approaches. In the management of both-column fractures, the S+IF approach is recommended if the S+IF approach and IL approach can achieve fracture exposure, reduction, and fixation.

## Data Availability

This article only includes summarized data from this study. Datasets are available from the corresponding author on reasonable request.

## Abbreviations

S+IF: Stoppa combined with iliac fossa
IL: Ilioinguinal
KL: Kocher-Langenbeck
ROM: Range of motion
CT: Computed tomography
3D: Three-dimensional
ASIS: Anterior superior iliac spine
LFCN: Lateral femoral cutaneous nerve
ACE: Anterior Combined Endopelvic
